# Effect of deferiprone or deferoxamine on right ventricular function in thalassemia major patients with myocardial iron overload

**DOI:** 10.1186/1532-429X-13-34

**Published:** 2011-07-06

**Authors:** Gillian C Smith, Francisco Alpendurada, John Paul Carpenter, Mohammed H Alam, Vasili Berdoukas, Markissia Karagiorga, Vasili Ladis, Antonio Piga, Athanassios Aessopos, Efstathios D Gotsis, Mark A Tanner, Mark A Westwood, Renzo Galanello, Michael Roughton, Dudley J Pennell

**Affiliations:** 1 CMR Unit, Royal Brompton & Harefield NHS Foundation Trust, Sydney Street, London SW3 6NP, UK; 2 National Heart & Lung Institute, Imperial College London, Guy Scadding Building, Dovehouse Street, London SW3 6LY, UK; 3 Division of Haematology/Oncology, Children's Hospital, 4650 Sunset Boulevard, Los Angeles, CA 90027, USA; 4 Thalassaemia Unit, Aghia Sophia Children's Hospital, Thivon & Papadiamantopoulou, Goudi, Athens 115 27, Greece; 5 Division of Paediatrics and thalassaemia centre, Department of Clinical and Biological Sciences, University of Torino, S. Luigi Gonzaga Hospital, Regione Gonzole 10, Orbassano 10043, Torino, Italy; 6 First Department of Internal Medicine, University of Athens Medical School, Laiko Hospital, 17 Agiou Thoma Street, Athens 115 27, Greece; 7 Department of Magnetic Resonance, Institute Euromedica-Encephalos, 3 Rizariou Street, Halandri, Athens 152 33, Greece; 8 Department of Cardiology, St Richard's Hospital, Western Sussex Hospitals NHS Trust, Chichester, West Sussex, PO19 6SE UK; 9 Department of Cardiology, The London Chest Hospital, Bonner Road, London E2 9JX, UK; 10 Department of Biomedical Science and Biotechnology, University of Cagliari, Ospedale Regionale per le Microcitemie. Via Edward Jenner, 09121 Cagliari, Italy; 11 UCL Cancer Trials Centre, University College London, 90 Tottenham Court Road, London W1T 4TJ, UK

## Abstract

**Background:**

Thalassaemia major (TM) patients need regular blood transfusions that lead to accumulation of iron and death from heart failure. Deferiprone has been reported to be superior to deferoxamine for the removal of cardiac iron and improvement in left ventricular (LV) function but little is known of their relative effects on the right ventricle (RV), which is being increasingly recognised as an important prognostic factor in cardiomyopathy. Therefore data from a prospective randomised controlled trial (RCT) comparing these chelators was retrospectively analysed to assess the RV responses to these drugs.

**Methods:**

In the RCT, 61 TM patients were randomised to receive either deferiprone or deferoxamine monotherapy, and CMR scans for T2* and cardiac function were obtained. Data were re-analysed for RV volumes and function at baseline, and after 6 and 12 months of treatment.

**Results:**

From baseline to 12 months, deferiprone reduced RV end systolic volume (ESV) from 37.7 to 34.2 mL (p = 0.008), whilst RV ejection fraction (EF) increased from 69.6 to 72.2% (p = 0.001). This was associated with a 27% increase in T2* (p < 0.001) and 3.1% increase in LVEF (p < 0.001). By contrast, deferoxamine showed no change in RVESV (38.1 to 39.1 mL, p = 0.38), or RVEF (70.0 to 69.9%, p = 0.93) whereas the T2* increased by 13% (p < 0.001), but with no change in LVEF (0.32%; p = 0.66). Analysis of between drugs treatment effects, showed significant improvements favouring deferiprone with a mean effect on RVESV of -1.82 mL (p = 0.014) and 1.16% for RVEF (p = 0.009). Using regression analysis the improvement in RVEF at 12 months was shown to be greater in patients with lower baseline EF values (p < 0.001), with a significant difference in RVEF of 3.5% favouring deferiprone over deferoxamine (p = 0.012).

**Conclusion:**

In this retrospective analysis of a prospective RCT, deferiprone monotherapy was superior to deferoxamine for improvement in RVEF and end-systolic volume. This improvement in the RV volumes and function may contribute to the improved cardiac outcomes seen with deferiprone.

## Introduction

Blood transfusions are standard therapy for patients with β-thalassaemia major (TM) and prevent death in childhood, but although clinical status and short term survival improve, each unit of blood contains about 200-250 mg of iron which the body cannot eliminate, which leads to long term iron accumulation. Patients treated only with blood transfusions may die in the second and third decades of life from the complications of iron overload, in particular heart failure [1,2]. Myocyte damage is related to the production of reactive oxygen species (ROS) formed as levels of labile iron rise, which cause oxidative damage to membranes and mitochondrial respiratory chain enzyme dysfunction [3,4]. Chelation therapy can reduce tissue iron levels and the incidence of cardiac complications, but patients at risk need to be accurately profiled for appropriate treatment. The cardiovascular magnetic resonance (CMR) relaxation parameter T2* is sensitive to storage tissue iron in haemosiderin because of the creation of field inhomogeneities by iron particles, and the clinical adoption of this technique is now widespread as a mainstay of cardiac iron overload assessment and treatment,[5-7] with important capability to predict future cardiac events,[8] and evidence of significant beneficial effects on cardiac mortality [9].

Deferoxamine was the first iron chelating agent for clinical use and became standard therapy in the 1970s. It is a large positively charged lipophobic molecule, is poorly absorbed by the digestive system and has a short plasma half life [10,11]. It is therefore administered subcutaneously using a portable syringe system usually overnight typically 5 times per week. This therapy can be very problematic with poor compliance, and a number of factors result in long-term cardiac iron accumulation with its use [12]. The second clinical iron chelator was deferiprone, which is a much smaller neutrally charged lipophilic molecule which allows good gastrointestinal absorption and cellular access [10,11]. The plasma half life is longer allowing oral administration with three doses per day. Direct comparison trials show that deferiprone has greater efficacy than deferoxamine for reducing myocardial iron loading and improving left ventricular (LV) systolic function [13,14]. However, there is a paucity of data related to the effects of these chelators on the right ventricle (RV), which is known to be an important independent predictor of outcome in dilated cardiomyopathy,[15] and ischaemic heart disease [16-18]. Recent papers have established the normal ranges for RV volumetric parameters for non-iron loaded TM patients and shown a significant relation between T2* and RV ejection fraction (RVEF), including a small percentage of patients with impaired RVEF but normal LVEF [19,20]. In order to identify and compare the effects of deferiprone and deferoxamine, we reanalyzed the CMR images for the LA16 trial, which was a randomized controlled trial (RCT) comparing the 2 drugs [14]. Our hypothesis was that deferiprone would improve RV function more than deferoxamine.

## Methods

The LA16 RCT consisted of 61 regularly transfused patients with TM from 4 centres in Greece and Italy [14]. All patients were previously treated with subcutaneous deferoxamine monotherapy. Inclusion criteria included a T2* between 8 and 20 ms and LVEF greater than 56% based on the lower normal limit for non-anaemic subjects from previously published data [21]. No patient had heart failure symptoms. Deferiprone was allocated to 29 patients (actual dose 92 mg/kg daily) whilst 32 patients were allocated to continue with deferoxamine therapy (dose of 43 mg/kg/day overnight for an average of 5.7 days per week). Written informed consent was obtained according to local ethics committee approval.

Iron loading and cardiac function was assessed using CMR. The T2* sequences were installed at the local CMR facilities, Athens (GE CVi) and Cagliari (GE Signa). The technique was validated by scanning phantoms of known T2* and testing intra-site reproducibility by scanning 5 patients twice at the local centre. The same patients were scanned at the reference site in London (Siemens Sonata) for inter-site reproducibility. A coefficient of variation (CV) ≤ 15% was defined as acceptable. Site inter-study variability was 2.4% for Cagliari and 3.5% for Athens. Comparison with the reference site yielded CVs of 1.6% and 9.7% respectively. Volumetric data were acquired using a steady state free precession sequence (FIESTA). A set of contiguous short axis cines were acquired to give complete coverage of both ventricles. Care was taken to place the basal slice parallel to the atrioventricular groove. Slice thickness was 8 mm with a spacing of 10 mm. No patient had a history of, and no CMR scan showed any features of pulmonary hypertension (normal pulmonary artery size, no right ventricular hypertrophy, no systolic septal flattening). Patients were scanned between 3 and 10 days post transfusion at baseline, 6 months and 12 months after entering the trial. T2* values and LV volumetric data were assessed previously using a CMR viewing and analysis software package CMRtools (Cardiovascular imaging solutions, London, UK) [14]. LV function was assessed in the RCT using an early version of the analysis package in which LV volumes are quantified manually using direct surface planimetry, and therefore for consistency we elected to use the same version of the software to analyze the RV thus eliminating the need for right sided valve tracking. Care was taken to include blood volume below the pulmonary valve. Basal regions with thin, non trabeculated muscle were considered atrial and were excluded. Papillary muscles were also excluded from the blood pool [22]. Although local blinding to the treatment arm was not possible due to the nature of drug administration (oral for deferiprone and subcutaneous for deferoxamine), all remote scan analysis performed at the core-lab in London was fully blinded to treatment. Study treatment was unblinded on completion of LV and T2* analysis. For the analysis of the RV volumetric data, all data sets were anonymized and analyzed in random order using the same analysis package by experienced operators blinded to treatment arm and LV response. To assess reproducibility, data-sets with an improvement in RVEF ≥ 5% at 12 months were reprocessed blindly.

### Statistical analysis

Continuous variables were compared using a paired t-test. T2* values were log transformed and changes expressed as geometric mean with coefficient of variation. Between groups comparison of drug effects were assessed using a repeated measurement ANOVA. Statistical significance was set at p < 0.05. To quantify reproducibility the coefficient of variance (CV) was calculated.

## Results

Full data on the LA16 study have been published including the patient demographics,[14] but the important trial summary findings are repeated here. In the deferiprone group 27 patients completed the study; 2 patients withdrew due to adverse events (elevated hepatic enzymes, in one case probably due to cytomegalovirus). In the deferoxamine group, 29 patients completed; 1 patient withdrew secondary to a reduction in LV function and 2 for personal reasons. The patient groups were well matched at baseline for cardiac T2*, LV volumes and function and RV volumes and function (table 1). Baseline RVEF was within the normal reference range for thalassaemia patients,[19] except for one patient in the deferoxamine group (RVEF 1% below the normal range). Patient compliance was similar for both groups. Myocardial T2* improved by 18% at 6 months (p < 0.001) and 27% at 12 months (p < 0.001) in the deferiprone arm. The LVEF rose by 2.0% from 69.7% at baseline to 71.7% at 6 months (p < 0.001) and by 3.1% to 72.7 at 12 months (p < 0.001). With deferoxamine therapy, T2* improved by 9% at 6 months (p = 0.003) and by 13% at 12 months (p < 0.001) but LVEF was unchanged being 68.4% at baseline and 68.7 at 6 months (+0.52%, p = 0.42) and 68.5 at 12 months (+0.32%, p = 0.66).

**Table 1:** Baseline values for cardiac volume and function parameters.

	Deferiprone	Deferoxamine	p
No. Patients randomized	28	32	
Age	25.1 ± 3.8	26.6 ± 4.7	0.33
Male sex (%)	15 (52)	16 (50)	0.99
Weight (kg)	57.7 ± 7.9	60.6 ± 13.2	0.30
Cardiac parameters			
Myocardial T2* (ms)	13.0 (32%)	13.3 (30%)	0.77
LVEDV (mL)	134 ± 32	132 ± 23	0.81
LVESV (mL)	43 ± 14	41 ± 13	0.51
LVEF (%)	69.7 ± 5.4	68.4 ± 4.9	0.34
RVEDV (mL)	122.5 ± 24.9	124.7 ± 27.7	0.75
RVESV (mL)	37.7 ± 11.7	38.1 ± 12.6	0.90
RVEF (%)	69.6 ± 5.2	70.0 ± 5.8	0.79
Biochemical markers			
Liver iron concentration (μg/L)	6.16 ± 6.0	6.32 ± 5.8	0.92
Serum ferritin (μg/L)	1791 ± 1029	2795 ± 2441	0.039
Haematology			
Transfusional iron (mL/kg/year)	152 ± 43.4	144 ± 44.4	0.53
Haemoglobin level (g/L)	105 ± 12.0	113 ± 11.9	0.023

Values are mean ± SD except for T2* where values are geometric mean with CV.

**Figure 1:**
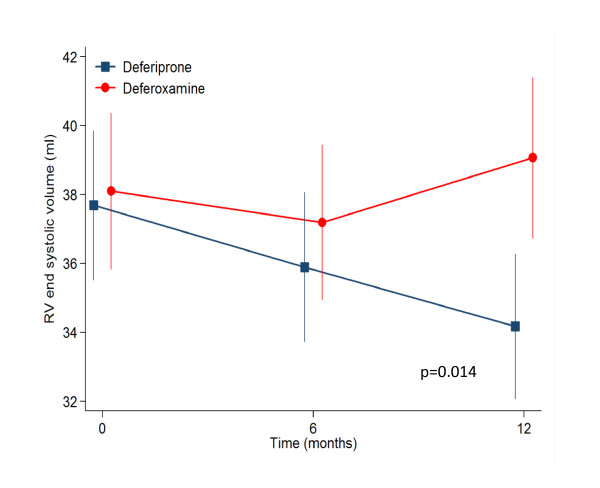
**The response in the 2 treatment arms for RVESV showed a significant improvement for patients treated with deferiprone, which was not seen with deferoxamine (12 month difference between drugs p = 0.014)**.

**Figure 2:**
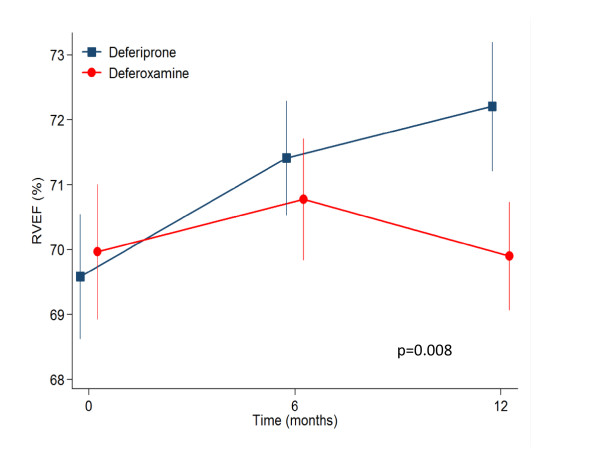
**The response in the 2 treatment arms for RVEF showed a significant improvement for patients treated with deferiprone, which was not seen with deferoxamine (12 month difference between drugs p = 0.008)**.

In the current analysis, the RV mean volumetric and T2* values are detailed in table 2. To summarise, in the deferiprone arm RV end-diastolic volume (EDV) was stable, RV end-systolic volume (ESV) decreased significantly from 37.7 to 34.2 mL at 12 months (p = 0.009); and RVEF increased from 69.6% to 72.2% (p = 0.001). For the patients on deferoxamine therapy, the changes in RV parameters from baseline to 12 months showed no significant difference. Analysis of between drugs treatment effects using a repeated measurement ANOVA (table 3) showed significant differences favouring deferiprone for the reduction of RV ESV (p = 0.009 at 12 months, Figure 1) and improvement in RVEF (p = 0.013 at 12 months, Figure 2). Non significant differences between drugs were found for RV EDV. With regression analysis, the change in RVEF was found to be inversely related to the baseline EF (p < 0.001) with a significant difference between drugs favouring deferiprone by a mean of 3.5% (95% CI 0.8 to 6.3%; p = 0.012). The reduction in RVESV over 12 months was also related to the baseline ESV value with borderline significance (p = 0.051), and there was a significant difference between drugs favouring deferiprone by a mean of 4.5 mL more than patients on deferoxamine (95% CI 1.2 to 7.8 mL; p = 0.009). Therefore the patients benefitting most from deferiprone treatment are those with the lower baseline values of RVEF. The CV for intra-observer study RVEF measurement was 2.4% at baseline and 2.0% at 12 months. There was no relation between change in RVEF and change in LVEF (r = 0.3, p = 0.9).

**Table 2:** RV volumetric parameters at baseline, 6 and 12 months (mean ± SD) in the 2 treatment arms.

		Baseline	6 Months	12 Months	p
Deferiprone					
	T2*, ms	13.0 (32)	15.4 (38)	16.5 (38)	< 0.001
	RVEDV, mL	122.5 ± 24.9	123.2 ± 26.0	121.3 ± 24.9	0.61
	RVESV, mL	37.7 ± 11.7	35.9 ± 11.7	34.2 ± 11.3	0.009
	RVSV, mL	84.7 ± 16.5	87.3 ± 16.5	87.1 ± 17.0	0.16
	RVEF, %	69.6 ± 5.2	71.4 ± 4.7	72.2 ± 5.3	0.001
					
Deferoxamine					
	T2*, ms	13.3 (30)	14.4 (37)	15 (39)	<0.001
	RVEDV, mL	124.7 ± 27.7	124.4 ± 26.2	128 ± 32.1	0.17
	RVESV, mL	38.1 ± 12.6	37.2 ± 12.5	39.1 ± 13.0	0.38
	RVSV, mL	86.7 ± 18.0	87.0 ± 15.5	88.9 ± 21.3	0.25
	RVEF, %	70.0 ± 5.8	70.8 ± 5.2	69.9 ± 4.6	0.93

The T2* values show the geometric mean and CV. The p value reflects the change from baseline to 12 months.

**Table 3:** Between drug effect on RV volumetric parameters showing a significant difference in RV ESV and RVEF favouring deferiprone.

	Treatment effect			
	Deferiprone-Deferoxamine			
	Mean	95% CI		p
EDV	-1.21	-4.44	2.03	0.47
ESV	-1.82	-3.27	-0.37	0.014
SV	0.64	-2.02	3.31	0.64
EF	1.16	0.30	2.01	0.008

## Discussion

RV volumetric and functional parameters have been difficult to measure using conventional imaging techniques due to the irregular geometry of the RV chamber, the size and quantity of the RV trabeculae, and the proximity of the RV to the chest wall which impairs echocardiographic assessment. CMR suffers less from these drawbacks because of its inherent 3D nature and high blood to myocardium contrast and is therefore considered to be the most accurate and reproducible technique for assessing RV volumes and EF [23,24]. Attention to correct definition of the basal slice during acquisition and subsequent analysis is however pivotal. The improved confidence of measuring RV volumes and function from CMR and other techniques has assisted the understanding the importance of the RV in cardiac disease. RVEF is an important predictor of outcome in dilated cardiomyopathy, which is both independent of and incremental to LV EF [15]. The predictive value of RV function has also been shown in congenital heart disease,[25-27] chronic systolic dysfunction,[28] and ischemic heart failure,[16,17,29,30] with RVEF being shown to be an independent predictor of outcome [16,17]. Accordingly, the effects of myocardial iron loading on RV function may be important in thalassaemia patients.

In the current study, we found a significant improvement in RVEF (increase) and RVESV (reduction) with deferiprone therapy. These improvements parallel the previously reported LV response [14]. There was no significant increase in RVEDV suggesting loading conditions did not play an important role. A flat RV response was seen in the deferoxamine group, which again mirrors LV behaviour. The between groups analysis showed superiority for deferiprone over deferoxamine for both the reduction in RVESV and the increase in RVEF. The magnitude of improvement in RVEF and reduction in RVESV were greater for patients with a higher ESV and lower EF at baseline. Interestingly neither LVEF nor RVEF improved significantly in the deferoxamine group despite the improvement in T2*. The cause for this difference in functional response is not fully understood, but the explanation may lie in the additional effects of deferiprone on restoring normal cardiac mitochondrial function,[31] possibly through effects on reducing reactive oxygen species [32].

There is little other data relating RV function changes with the iron chelators, but a recently published abstract relating to a longitudinal trial of the efficacy of deferasirox in myocardial siderosis,[33] showed a significant improvement in myocardial iron levels with an improvement in RVEF at 1 year, but no change in LV function at 1,[34] 2,[35] and 3 [36] years of follow up. The significance of this discrepancy between RV and LV response to deferasirox is not currently clear, though it is possible that the RV response is an early signal of myocardial iron clearance as LV compliance and filling pressure improves.

### Limitations

Data acquisition for this study was originally designed to assess the change in T2* and LV functional parameters in response to therapy. Therefore no RV long axis images were obtained to construct 3-dimentional models for volumetric analysis, but the requirement for this was removed by using direct manual planimetry for quantitative analysis of RV volumes. Pulmonary arterial pressure was not systematically measured using echocardiography of the tricuspid regurgitant jet, but here was no CMR evidence of raised pulmonary artery pressure in our patients, and pulmonary hypertension is rare in well treated thalassaemia major [37]. Direct RV measurement of T2* would have been interesting in this population to compare with changes in RV volumes and function, however, it is challenging to measure T2* in the thin wall of the RV and this was not attempted in the randomized controlled trial.

## Conclusions

This study has shown that RVESV decreased and RVEF improved with deferiprone monotherapy and this beneficial response was superior to deferoxamine. RV volumetric and function parameters have in the past been neglected when reporting the efficacy of iron chelators for myocardial iron overload, and may have independent prognostic importance, as they do in other cardiac conditions with impaired cardiac function.

## Competing interests

GCS is a consultant to Novartis and has received honoraria from ApoPharma. FA has received honoraria from Novartis. JPC has received speaker's honoraria from Novartis, Swedish Orphan and ApoPharma. VB is a consultant for ApoPharma. VL has received grant support, consulting fees and lecture fees from ApoPharma and Novartis. AP has received honoraria and research funding from Novartis. RG has served on speakers' bureaus and received research grants from Novartis and ApoPharma. DJP is a consultant to and has served on advisory boards and speakers' bureaus for Novartis, ApoPharma, and Siemens; has received research funding from Novartis; and is a director and stockholder for Cardiovascular Imaging Solutions.

## Authors' contributions

GCS and FA participated in study design, data analysis and interpretation and manuscript drafting. JPC and MHA performed additional data analysis. VB, MK, VL, AP, AA, EDG, MAT and MAW Served as investigators on the original trial. MR performed statistical analysis. DJP conceived of the study and was responsible for the final manuscript draft. All authors read and approved the final manuscript.
